# Supplementary material for: Patterns of Intron Gain and Loss in Fungi
Source: PLoS Biol. 2004 Nov 30;2(12):e422. doi: 10.1371/journal.pbio.0020422 (PMC532390; doi:10.1371/journal.pbio.0020422)
Supplement: Table S1 — Also available at http://genes.mit.edu/NielsenEtAl/. (4.3 MB ZIP). [file pbio.0020422.st001.zip › NielsenEtAl/html/191.html]

AN0070.1.NCU05692.1.MG10973.1.FG02707.1


```
 CLUSTAL W (1.82) Multiple Sequence Alignments - Introns Inserted


Sequence 1: NCU05692.1	220 aa
Sequence 2: FG02707.1	175 aa
Sequence 3: MG10973.1	194 aa
Sequence 4: AN0070.1	183 aa
Alignment Length: 233 aa
Number Identitical Residues: 53 aa
Alignment Score (without introns) 2484


MG10973.1 	MTTQGGDDTVPLPALPPQQLSTSSKVPSKLPAIAALPGLDDASLIGTLDLLFEASPDLHA
NCU05692.1	---MSSHTHTHSTHLNPEATPF-------LPAITSLPTLPDAALISTLDLLFEPSPDLHA
FG02707.1 	-----------MTLLPPKE----------------LRIANETEQIKTLDLLFEPSPAIHS
AN0070.1  	-----------MPSLPQPS---------------SLRELPQEEKFQVLDTLFESSPELHT
          	            . *                   :*    :   : .** ***.** :*:

MG10973.1 	LAVPVARSMTFTSYRDLIESIRSTLLSVQA------TVHADPVAR-----KPLLGILAAH
NCU05692.1	LALPTLRTISFSSYDDLISTLRDELLAIADAVALAAADPSVPQIEKENIQRPLHAILGSH
FG02707.1 	TLIPVLRESEYTSYPELIDACRSRLVSLAS-----SSSPTNPDAT-------LLSILGSH
AN0070.1  	LMAPILANQTFSSYASLIDAVGGRMSALSA--------ANSPTDR-----DILVGILGSH
          	   *      ::** .**.:  . : ::             *          * .**.:*

MG10973.1 	PRLGE---RKQP-VSDQSKAEQASLQGEA------EKLATLNAEYEAKFPGMRYV~VFVN
NCU05692.1	PRLGE---AKKEKLSAQSRKEQAHLNSGGDSEEVKEELRRLNKMYEETFPGLRYV~VWVN
FG02707.1 	PRLGA---KKVE--SAQSAAEQANLQGQG------EELAKLNQEYEEKFPGLRYV~VFVN
AN0070.1  	PRLGQPKGTAAEHLSELSKREQANLNTGA--EEQAEKLRLLNAEYEAKFPGLRFV2TFVN
          	**** ...      *  *  *** *:  .  ..  *:*  **  ** .***:*:* .:**

MG10973.1 	GRSRDIIMENMRARIDRGDMNAEEQDAIN0-------KRYYNII-----------
NCU05692.1	GRGRGEVMADMRRRIARGDLRAEEREGIQ0AMCDIAADRAAKLLKSAQEAVEHSA
FG02707.1 	GRGRPEIMENMKARISRGVFSKEVAEALQ0AMCDIAKDRASKLDAKL--------
AN0070.1  	GRSRDVIMEEMRARIDRGDTEREIEDIIQ0LEYSLDWRKIAGASEK---------
          	**.*  :* :*: ** **    *  : ::    .    :      .
```
